# Identifying Biomarkers for Atherosclerosis *via* Gene Expression and Biological Networking

**DOI:** 10.2174/011573403X340118241113025519

**Published:** 2025-01-03

**Authors:** Sangeeta Chhotaray, Soumya Jal

**Affiliations:** 1 School of Paramedics and Allied Health Sciences, Centurion University of Technology and Management, Bhubaneswar, Odisha, India

**Keywords:** Atherosclerosis, cardiovascular disease, biomarker, KEGG, STRING, hyperlipidemia

## Abstract

**Introduction:**

Atherosclerosis is a chronic disease caused by the accumulation of lipids, inflammatory cells, and fibrous elements in arterial walls, leading to plaque formation and cardiovascular conditions like coronary artery disease, stroke, and peripheral arterial disease. Factors like hyperlipidemia, hypertension, smoking, and diabetes contribute to its development. Diagnosis relies on imaging and biomarkers, while management includes lifestyle modifications, pharmacotherapy, and surgical interventions. Computational biology is transforming biological knowledge into clinical practice by identifying biomarkers that can predict clinical outcomes. This involves omics data, predictive modeling, and data integration. Statistical analysis-based methods are also being developed to develop and integrate methods for screening, diagnosing, and prognosing atherosclerosis.

**Methodology:**

The present work aimed to uncover critical genes and pathways to enhance the understanding of the mechanism of atherosclerosis. GSE23746 was analyzed to find differentially expressed genes (DEGs) using 19 control samples and 76 atherosclerotic samples.

**Results:**

A total of 76 DEGs were identified. Analysed DEGs using Gene Ontology (GO) and Kyoto Encyclopaedia of Genes and Genomes (KEGG) to generate enrichment datasets. A Protein-protein Interaction (PPI) network of DEGs was created utilizing the Search Tool for the Retrieval of Interacting Genes (STRING).

**Conclusion:**

Ten hub genes, namely EGR1, PTGS2, TNF, NFKBIA, CXCL8, TNFAIP3, CCL3, IL1B, PTPRC, and CD83, were found to be significantly linked to atherosclerosis. Furthermore, the metabolic pathway analysis through KEGG and STRING provides potential targets for therapeutic interventions through HUB genes to diagnose the illness at an early stage, which aids in the reduction of cardiovascular risk. From risk factor profiling to the discovery of novel biomarkers, several components such as phospholipids, ANGPTL3, LCAT, and the protein-encoded OCT-1 gene, play a vital role in crucial processes. These compounds are potential therapeutic targets for early diagnosis of atherosclerotic lesions and future novel biomarkers.

## 1 INTRODUCTION

Atherosclerosis is a disease characterized by inflammation, and it involves the participation of various chemokines. These molecules play various roles, including recruiting circulating inflammatory cells and activating inflammatory and pro-thrombotic processes. Ultimately, these processes contribute to the occurrence of atherosclerosis-related events such as coronary artery disease, stroke, and peripheral arterial disease, which are substantial global health concerns [1, 2]. The high prevalence of atherosclerosis and related cardiovascular disorders necessitates research into the causes and risk factors contributing to their development. The formation of atherosclerotic plaque depends on the uptake of cholesterol from the bloodstream by cells beneath the endothelium. Hypercholesterolemia is a well-known determinant of atherosclerosis, and cholesterol-lowering therapy is commonly utilized in clinical practice to treat cardiovascular disease. Many molecular pathways have been linked to the development of cardiovascular events, involving variables like inflammation, oxidative stress, and others. Detecting biological markers linked to inflammation and oxidative stress can help healthcare providers evaluate therapy effectiveness and develop new pharmaceutical strategies for persons at high cardiovascular disease risk [3]. It is now understood to be a chronic inflammatory disease with several immune system components (both innate and adaptive), which influence lesion onset and development and can lead to life-threatening thrombotic consequences [4].

Comprehending the fundamental elements of inflammatory processes is essential for uncovering the complex underlying mechanisms involved in the development of atherosclerosis. The medical community is seeking a more cost-effective therapy due to the high cost of present treatments and their limited effectiveness. Understanding the causes and mechanisms of atherosclerosis will help in its treatment and prevention. Discovering novel indications linked to an increased susceptibility to atherosclerotic vascular disease might expand our understanding of the disease's fundamental causes and aid in the development of more effective preventative and treatment approaches [5].

### 1.1 Development of Atherosclerosis and the Formation of Fatty Streaks

**Fig. (1):**
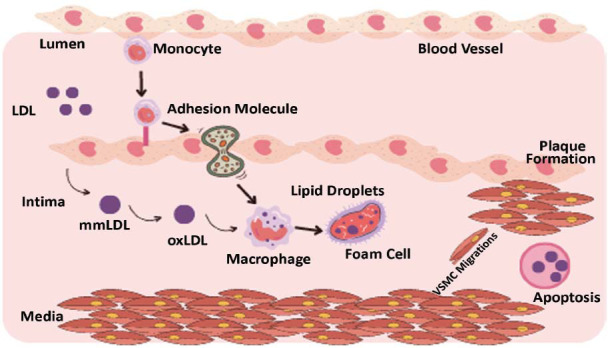
Mechanisms involving cells and molecules that cause atherosclerosis in the arterial wall. [Low-density lipoproteins (LDL) cholesterol can permeate the arterial wall and gather within the subendothelial space. These LDL cholesterols undergo oxidation to elicit an inflammatory reaction. The injured area attracts inflammatory cells like monocytes and macrophages. Foam cells arise when these cells take in the oxidized LDL cholesterol. Foam cells and lipid deposits combine to form fatty streaks. Formation of the atherosclerotic plaque involves smooth muscle cells undergoing migration from the arterial media (middle layer) to the arterial intima (inner layer), which is the inner layer of the arterial wall. Subsequently, these cells undergo proliferation, leading to the formation of plaque].

Atherosclerosis initiation commences with damage to the intima. This injury can result from various conditions such as hypertension, smoking, hypercholesterolemia, diabetes, and inflammation. Endothelial injury increases the permeability of the endothelium to lipids, such as low-density lipoprotein (LDL) cholesterol. Over time, these cholesterol particles get oxidized, leading to an inflammatory reaction by the body's immune cells. Macrophages, a kind of immune cell, ingest oxidized LDL cholesterol, resulting in the development of foam cells (Fig. **1**). Foam cells, calcium deposits, and other cellular waste combine to create a plaque in the intima [6]. As the plaque accumulates, it constricts the artery, so limiting the circulation of blood to the organs and tissues that receive blood by that artery. These issues can arise, depending on the specific location and severity of the plaque. Plaque rupture can set off the process of blood clot production, which in turn can block the artery entirely, leading to a heart attack or stroke. Due to its widespread prevalence, there is an urgent need to comprehend the disease's origins and make progress in managing it. The majority of these diseases are diagnosed clinically, but numerous supplementary methods can help confirm or rule out the clinical diagnosis. Among these supplementary methods, the function of biomarkers is becoming significantly important [7].

### 1.2 Role of Biomarker in Atherosclerosis

Biomarkers have been defined in earlier days as observable, measurable, and quantifiable qualities that serve as indications of physiological, pathological, or pharmacological processes or therapeutic responses. However, functional biomarkers can detect pathological alterations years before the onset of clinical symptoms, which could help direct treatment action [8]. Recently, atherosclerosis biomarkers have emerged as crucial tools for the early diagnosis of cardiovascular disease and stroke [9]. They offer a potent method for comprehending the magnitude of cardiovascular illness. They have a wide range of applications, including screening, diagnosing, predicting outcomes, and monitoring treatment progress. The disciplines of bioinformatics, genomics, proteomics, and metabolomics have greatly revolutionized the quest for possible markers that can offer useful insights into the different stages of atherosclerosis. Before implementing them in practice, it is crucial to comprehend the particular uses, establish consistent analysis methods, assess performance attributes, and ascertain the additional value and efficiency of several markers for specific medical applications [10].

### 1.3 Classification of Atherosclerotic Biomarker

**Fig. (2):**
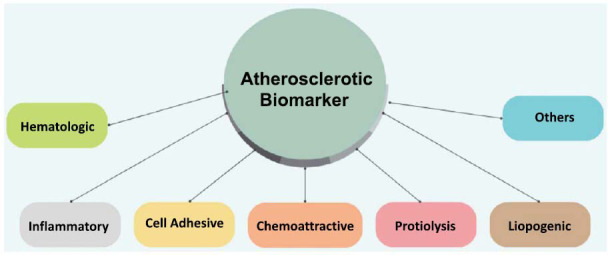
Several distinct categories of atherosclerotic biomarker.

Biomarkers offer a potent method for comprehending the range of cardiovascular diseases. They are utilized in various fields, including screening, diagnosis, prognostication, recurrence prediction, and therapeutic monitoring. These molecules are measurable indicators that can be utilized to evaluate the presence, progression, or lethal effect of a disease. In the case of atherosclerosis, several Biomarkers have the potential to be utilized for evaluating the disease condition (Fig. **2**).

### 1.4 Early Validated Biomarkers

#### 1.4.1 Inflammatory Biomarkers

Inflammatory biomarkers linked to atherosclerosis serve as indicators of the ongoing inflammation occurring within the arterial walls. This Inflammation is a crucial factor in the development and progression of atherosclerotic plaques. Monitoring these biomarkers can offer valuable insights into the level of inflammation and aid in evaluating the threat of cardiovascular events. Additionally, to their important involvement in inflammation, the protein mediators known as cytokines are involved in a wide various other physiological systems. Interleukins (ILs), tumor necrosis factors (TNFs), and interferons (IFNs) transforming growth factors (TGFs), colony-stimulating factors (CSFs), and various chemokines are just a few of the numerous kinds of cytokines. Endothelial cell (EC) activation by cytokines causes endothelial dysfunction and elevation of adhesion molecules and chemokines, which enhance immune cell migration into atherosclerotic sites [11]. Monitoring these inflammatory biomarkers, along with traditional risk factors like cholesterol levels and blood pressure, can provide a more comprehensive assessment of cardiovascular risk and guide treatment strategies aimed at reducing inflammation and preventing atherosclerotic complications.

#### 1.4.2 Cell Adhesive Biomarkers

Atherosclerosis is mostly due to cell adhesion molecules (CAMs), which mediate contacts between different types of cells within the artery walls. These cells encompass endothelial cells, immunological cells, and smooth muscle cells. Atherosclerotic plaque development, inflammation, and the immunological response all rely on these relationships. Afterward, in an inflammatory reaction, leukocytes transmigrate into the intima *via* the endothelial layer [12]. As a preliminary step in the “adhesion” process, a selectin family of adhesion proteins mediates the tethering and rolling of blood cells along the surface of the endothelium monolayer [13]. Over the past few decades, a large number of cell adhesion molecules (CAMs) have characterized, and analyzed how they contribute to various processes. It is perceivable that CAMs have a profound effect on various processes, such as cell-cell and cell-matrix interactions, cell migration, cell cycle, signal transduction, and morphogenesis in both embryonic and adult tissues [14]. Four distinct families of CAMs have been identified during the processes of development and tissue regeneration. These families include the cadherins, the selectins, the integrins, and the immunoglobulin CAM (Ig-CAM) superfamily. CAMs perform a crucial characteristic in engaging immune cells, including monocytes and T cells, to the arterial wall. Once there, these molecules trigger and sustain inflammation. The inflammatory response performs a pivotal role in the development of atherosclerosis and the formation of plaque. Cell adhesion molecules have the potential to function as biomarkers for evaluating the advancement and seriousness of atherosclerosis. Furthermore, these molecules serve as potential targets for therapeutic interventions that aim to decrease inflammation and mitigate the associated risks of cardiovascular events.

#### 1.4.3 Chemoattractant Biomarker

**Fig. (3):**
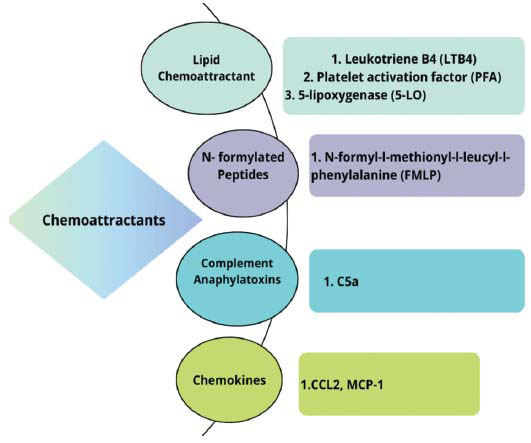
Exhaustive enumerations of diverse chemoattractant categories with supporting examples.

Chemoattractant biomarkers associated with atherosclerosis, indicate those molecules that can attract immune cells, specifically monocytes, and leukocytes, to the specific location of inflammation within the arterial walls (Fig. **3** and Table **1**). Biomarkers are essential in the recruitment of immune cells, which are responsible for the development and advancement of atherosclerotic plaques. Among all immune cells, neutrophils respond to a wide variety of chemoattractants that are released from a variety of sources. They belong to a wide variety of chemical families, including lipids, N-formylated peptides, complement anaphylotoxins, and chemokines [15].

#### 1.4.4 Proteolysis-related Biomarker

Proteolytic enzymes may not cause atherosclerosis directly, but they do contribute to plaque development and destabilization in some ways. Proteolytic enzymes like matrix metalloproteinases (MMPs) degrade extracellular matrix components within plaques, making them more susceptible to damage and rupture. These MMPs are significantly elevated in comparison to normal levels during acute coronary emergencies [22].

#### 1.4.5 Lipogenic Biomarker

In addition to age, cholesterol along with LDL levels are highly significant determinants for future cardiovascular events. More than a century ago, the term “atheroma” was coined to refer to a yellow fatty substance, often known as plaque (later recognized as cholesterol), and indicated the role of lipids in atherosclerosis [23]. This epidemiologic link sparked decades of research into cholesterol, cholesterol-trafficking lipoproteins, and the cellular and molecular mechanisms that regulate the metabolism of cholesterol and are associated with atheroma formation [24]. Numerous lipid indicators are strongly linked to atherosclerosis, including LDL, HDL, VLDL, *etc*.

#### 1.4.6 Hematologic Biomarker

Hematological biomarkers associated with atherosclerosis are indicators obtained from hematological (blood) examinations. These biomarkers offer valuable information about the processes of inflammation, clotting, and metabolism of lipids that participate as pivotal characters in the development and evolvement of atherosclerotic plaques. Leukocytosis, or an excess of white blood cells, has been linked to inflammation that occurs due to atherosclerosis. In particular, research has also connected atherosclerotic lesion risk to higher levels of neutrophils and monocytes. Although platelets are important for blood coagulation, they can also contribute to atherosclerotic plaque development [25]. These hematological biomarkers provide valuable information about the underlying biological processes that contribute to atherosclerosis.

#### 1.4.7 Different Haematological Components as Atherogenic Markers

##### 1.4.7.1 WBC

The effect of leukocytes on the instability of atherosclerotic plaques is pivotal in the pathophysiological aspect. Initially, leukocytes invade endothelial cells and then activate the tunica intima. They cause microvascularity to form, which weakens the plaque and increases the risk of it rupturing [26].

##### 1.4.7.2 Red Cell Distribution Width

The red cell distribution width (RDW) component of a routine CBC measures the extent to which red blood cell volume varies. Anisocytosis is characterized by an increase in RDW. Patients with hemolysis have been observed to have elevated levels of RDW [27]. Rather than this, atherosclerosis in the brain and peripheral artery disease have both been associated with high RDW [28].

##### 1.4.7.3 Platelets & Plaquetogram

Platelets are a form of blood cell that aids in the process of blood coagulation. Platelet counts indicate a dynamic balance of numerous concurrent events, such as the coagulation process, changed capillary permeability, and inflammatory cascades [29]. It aids in the development of blood clots, which can block atherosclerotically constricted arteries [30]. Therefore, it is imperative to analyze platelets both qualitatively and quantitatively. The electronic counters' ability to assess a wide range of quantitative and qualitative platelet parameters justifies the use of the word plaquetogram in place of the more traditional hemogram. Platelet count (PLT), mean platelet volume (MPV), platelet distribution width (PDW), cross-linked platelets (RT), and platelet-cell ratio (P-LCR) were among the parameters assessed through plaquetograms [31]. Platelets have also been linked to the activation and coordination of endothelium, regardless of their part in the broader (systemic) inflammatory response [32].

## 2 METHODOLOGY OF ANALYSIS

### 2.1 Microarray Dataset Collection

The cardiac RNA expression dataset was obtained from the online GEO database. The original search employed the terms “atherosclerosis” and “*Homo sapiens*”. We select GPL2700 platform datasets as model sets. The entirety of the data is accessible to the public for online retrieval, and the research conducted by the scientists did not entail any experimentation on either humans or animals.

### 2.2 Data Processing and Identification of DEGs

The GEO2R tool, available at https://www.ncbi.nlm.nih.gov/geo/geo2r/?acc=GSE23746, was employed to conduct a comparative analysis of gene expression profiles in healthy people and atherosclerotic tissues. The Benjamini-Hochberg approach was applied to ascertain the false discovery rate, while the modified *P*-value was utilized to mitigate the probability of false positive mistakes. The DEGs were selected based on a *P*-value of less than 0.05 after adjustment and an absolute log fold change (FC) of at least 0.5.

### 2.3 Gene Ontology Enrichment Analysis

The use of GO is a common method for identifying distinctive biological features in high-throughput transcriptome data. This analysis was utilized to investigate the function of DEGs. This section examines the biological processes (BP), cellular components (CC), and molecular functions (MF) of genes with variable expression patterns [33]. KEGG is an extensive database that oversees and maintains data regarding genomes, biological pathways, diseases, pharmaceuticals, and chemical compounds [34]. The Database for Annotation Visualisation and Integrated Discovery (DAVID) and shinyGO tools are used to analyze and interpret the functions of many proteins. It also helps in integrating this information to understand the GO and KEGG pathways of identified DEGs [35].

### 2.4 Construction of Protein-protein Interaction (PPI) Networks

Protein interaction data was retrieved from STRING (https://string-db.org) after the putative targets of atherosclerosis and related diseases were entered and the criteria “minimum required interaction score 0.4” was specified. Using the degree values of the nodes, the primary objectives were chosen [36]. The PPI network of the overlapping DEGs was examined using it which is a comprehensive and influential online resource for analyzing the relationships between predicted and experimental protein interactions.

### 2.5 Evaluation of Specific KEGG Pathways in Detail to Identify Potential Targets

To learn more about the biological processes and pathways associated with atherosclerosis and conduct visual analysis, we entered the intersecting targets of atherosclerosis and its related conditions into KEGG analysis [37].

#### 2.5.1 OCT-1 (Octamer-binding Transcription Factor 1)

**Fig. (4):**
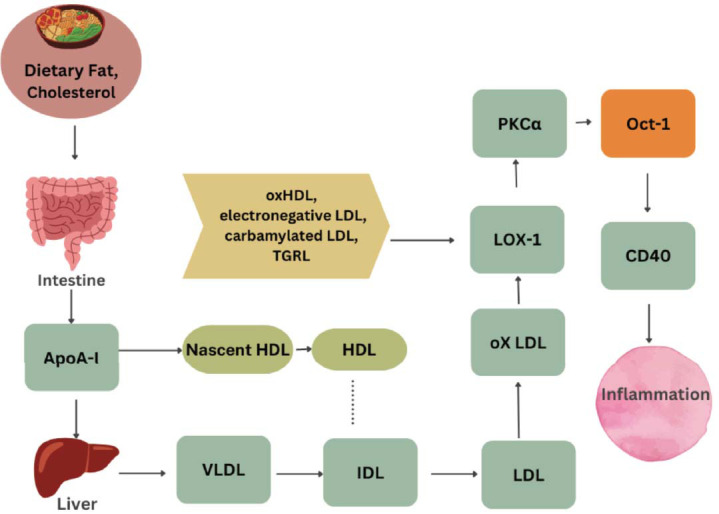
An exploratory summary of the biological mechanism leading to CD40-mediated inflammation in atherosclerosis. [OCT-1 has been found to interact with several inflammatory and immune-related genes. It can regulate the expression of genes involved in immune responses, and production of cytokines, and leads to CD40-mediated inflammatory processes in atherosclerosis].

Genes belonging to the homeobox family have a highly conserved DNA-binding domain and are involved in controlling proliferation, differentiation, and migration across a wide range of cell types. These genes can affect lipid metabolism and atherosclerosis-causing processes, including inflammation. They might control the gene expression related to lipid management and immunological response, both of which are crucial stages in the atherosclerotic process [38, 39]. The POU subfamily of homeodomain proteins includes the Oct-1 gene. These Oct1 gene functions contribute to the progression of atherosclerosis by facilitating inflammation and activating a variety of cellular processes (Fig. **4**).

The KEGG database was used for the systematic study of gene networks and their roles in molecular processes [34]. Genes related to lipid metabolism, inflammation, oxidative stress, and vascular function are all linked to atherosclerosis. Oct-1 (octamer-binding transcription factor 1) is a transcription factor that participates in the formation of atherosclerosis. It is important for a cascade of biological events that culminate in the production of CD40, an inflammatory molecule implicated in atherosclerosis. This analysis data suggests this Involvement as an immunological response that is facilitated by CD40.

#### 2.5.2 Oxidised Phospholipids

##### 2.5.2.1 Biological Prospective of Oxidized Phospholipids

The involvement of lipid peroxidation in disease is a prominent subject of research in the twenty-first century. Oxidized phospholipids, found in diseased conditions, often exhibit higher amounts compared to normal physiological states, attracting interest in this area [40]. The diseases that are associated with oxidised phospholipids and their products are hyperlipidemia, atherosclerosis, lupus, and arthritis [40]. It has also been shown that oxidised phospholipids cause endothelial cell inflammation. The accumulation of chemicals that bind to leukocytes on the endothelium surface, which monocytes use for adhesion, and the synthesis of chemotactic factors both rise during this inflammatory response [41].

##### 2.5.2.2 Role of Oxidised Phospholipids in Atherosclerosis

**Fig. (5):**
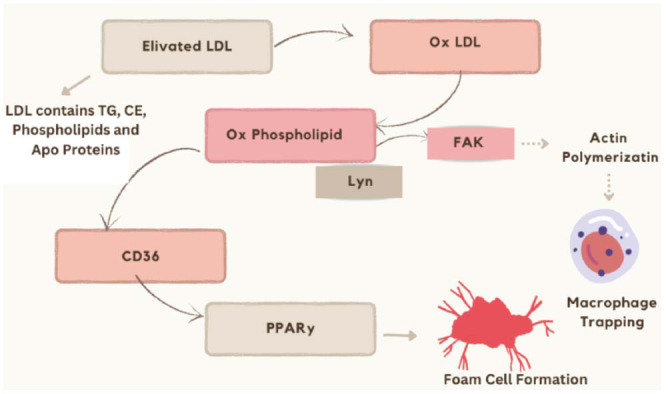
Phospholipid-mediated pathways result in the production of foam cells. [Low-density lipoprotein (LDL) particles undergo diverse alterations within the arterial wall. One of the primary alterations involves the process of oxidation, which leads to the production of oxidizedoxidized phospholipids (oxPL) within the low-density lipoprotein (LDL) particle. Oxidized phospholipids (oxPL) are recognized by scavenger receptors located on the surface of macrophages. Scavenger receptors, such as CD36, can form foam cells by activating peroxisome proliferator-activated receptorγ (PPARγ). Furthermore, when working with tyrosine-protein kinase Lyn, it initiates the activation of focal adhesion kinase, resulting in the entrapment of macrophages by actin polymerization].

Multiple phases of atherosclerosis, including lipoprotein metabolism, foam cell formation, and plaque stability, involve phospholipids. Cholesterol and phospholipids are both carried around the body by LDL particles. Phospholipid-rich LDL particles are highly prone to oxidation, which contributes to the development and advancement of atherosclerosis (Fig. **5**). Oxidised LDL particles in the artery wall are taken up by immune cells, primarily macrophages, and cause an inflammatory reaction [42]. Like other chronic diseases, atherosclerosis is linked to the buildup of oxidized phospholipids (some of which can also be generated enzymatically), which, along with different hydrolyzing enzymes, play a role in atherogenesis control [43].

##### 2.5.2.3 Mechanism of Oxidized Phospholipids Related Inflammatory Signaling in Atherosclerosis

Recent research has demonstrated that oxidized phospholipids (oxPLs), generated by LDL oxidation and Lp(a)-attached apoptotic cell membranes, build up in areas of persistent inflammation, such as atherosclerotic plaque, and help to accelerate the disease. Through interactions with immunological and vascular cells, they control intracellular signaling pathways, stimulate the expression of different cytokines, and influence cell behavior such as metabolic status, proliferation, apoptosis, *etc.* [44]. In this research work, KEGG is used while conducting pathway analysis.

Based on the findings of this pathway analysis, it is evident that CD36 exerts a major influence on the development of foam cells. Initially, it was believed that CD36 was a scavenger receptor rather than a signaling receptor. It is now understood that oxidized phospholipid activation of CD36 has significant signaling effects on both foam cell generation and differentiation [45]. Rather than this, many of the cellular activities linked to inflammation and atherosclerosis can be initiated or modulated by biologically active oxidized phospholipids. Inflammatory signals are also generated when these molecules, which can be generated by either enzymatic or non-enzymatic processes, contact specific receptors on the surfaces of different types of cells, like macrophages, and initiate macrophage trapping [46].

#### 2.5.3 Angiopoietin-like 3 Proteins

##### 2.5.3.1 Biological Perspective of Angiopoietin-like 3 Proteins (ANGPTL3)

**Fig. (6):**
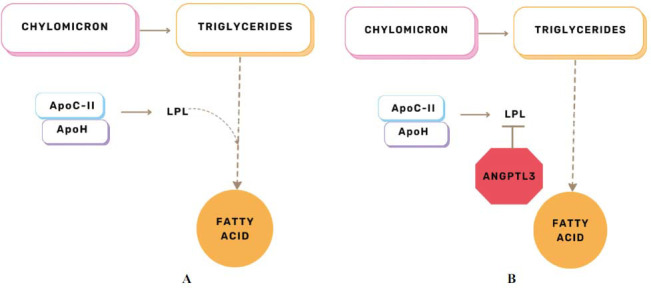
**A**) Conversion of TG to FA in the absence of ANGPTL3. **B**) Conversion of TG to FA in the presence of ANGPTL3.

Atherosclerosis and coronary heart disease (CHD) both have hyperlipidemia as a key risk factor. And the angiopoietin-like 3 proteins (ANGPTL3) have become a significant governing entity of lipoprotein metabolism in humans. It has become a potential molecular target for reducing cardiovascular risk due to its ability to regulate three important lipid traits: LDLC, high-density lipoprotein cholesterol, and TG [47]. However, lipoprotein lipase (LPL), an enzyme linked to the capillary endothelium that catalyzes the hydrolytic cleavage of triglycerides into fatty acids, is inhibited by ANGPTL3, making it a significant regulator of plasma triglyceride levels (Figs. **6A**, **B**). Following the hydrolysis of TRG by LPL, the residual chylomicrons and VLDL are eliminated *via* certain hepatic receptors, and the free fatty acids (FFAs) are absorbed by peripheral tissues as energy sources [48]. Therefore, this procedure is crucial to the metabolism of lipids [49, 50].

##### 2.5.3.2 Role of ANGPTL3 in the Formation of Atherosclerosis

The modulation of TG-rich lipoproteins such as chylomicrons and VLDL is mediated by ANGPTL3, which is largely formed by the liver. It works as an inhibitor of lipoprotein lipase (LPL), an enzyme that degrades triglycerides to these lipoproteins and slows down the clearance of those molecules from the bloodstream [51]. Additionally, it is evident from the KEGG study that these processes prevent triglyceride breakdown, which raises the levels of TG and chylomicrons. Increased TG levels result in the formation of atherosclerosis.

#### 2.5.4 Lecithin-cholesterol Acyltransferase

##### 2.5.4.1 Biological Prospective of Lecithin-cholesterol Acyltransferase

**Fig. (7):**
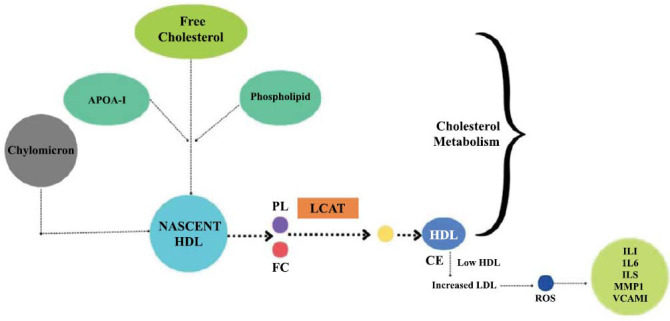
RCT mechanism. [During the process of lipolysis, chylomicrons undergo breakdown, resulting in the delivery of various products into the bloodstream. One of these products is free cholesterol. Apolipoproteins, especially apolipoprotein A-I (apoA-I), play a crucial role in picking up cholesterol molecules. ApoA-I is a vital component of high-density lipoprotein (HDL). The newly formed HDL particles are formed initially by the interaction between apoA-I and the cholesterol molecules that are released. Nascent HDL particles continue to acquire additional cholesterol and other lipids, such as phospholipids. As they do so, they mature into larger, more complex HDL particles. The maturation process involves interactions with enzymes, such as lecithin-cholesterol acyltransferase (LCAT), which esterify free cholesterol within the HDL particle, converting it into cholesterol esters. When the HDL level gets decreased the LDL level increases and interacts with the ROS which results in the formation of other regulated markers like IL1, IL6, MMP1 *etc*].

Lecithin-cholesterol acyltransferase (LCAT) is a glycoprotein that aids in the movement of acyl groups from lecithin to cholesterol. It has a crucial function in lipid metabolism, specifically in the transport and breakdown of cholesterol in the body. It has a function in the conversion of cholesterol into esters. The liver is the primary site of cholesterol synthesis, although minor amounts are also produced in the testes and astrocytes in the brain [52, 53]. The reverse cholesterol transport mechanism is facilitated by this protein, which is important for the proper development, interconversion, and rearrangement of all lipoprotein classes [54]. Reverse cholesterol transport (RCT) is an anti-atherogenic process in which high-density lipoprotein (HDL) excludes excess cholesterol from the cells and supplies it to the liver for elimination (Fig. **7**). Growing evidence supports a link between elevated LCAT activity and smaller LDL particles and its role in cholesterol esterification within HDL particles which contribute to the protective mechanisms against atherosclerosis through processes like reverse cholesterol transport and anti-inflammatory effects [55]. Hence monitoring LCAT activity can help identify individuals who are more likely to be susceptible to atherosclerosis [56].

##### 2.5.4.2 Mechanism of Action

The liver plays a crucial function in regulating cholesterol levels and preventing the accumulation of cholesterol inside the walls of arteries. This is particularly crucial in preventing the formation of atherosclerosis. The RCT mechanism is responsible for carrying out this process. The RCT route begins with the synthesis of apoAI in the liver or gut, which is subsequently delivered into the plasma [55]. Cholesterol esterification by LCAT causes the ndHDL particle to undergo a maturation process to convert into a more mature HDL particle [57, 58]. Therefore, the risk of developing atherosclerotic cardiovascular disease is strongly and independently inversely linked with plasma concentrations of HDL-C [59].

This KEGG analysis route demonstrates RCT, which is the process of transferring extrahepatic cholesterol, including cholesterol in atherosclerotic plaques, to the liver for elimination. To produce fully developed HDL particles that are involved in the cholesterol ester transfer protein (CETP) reaction and ultimately transport cholesterol to the liver, cholesterol esters produced by LCAT are stored in the core of the HDL particle. This leads to a progressive rise in the size of HDL particles. Consequently, the activation of LCAT leads to elevated levels of HDLC [60].

## 3 RESULTS AND DISCUSSION

### 3.1 Differentially Expressed Genes in Samples with Atherosclerosis Compared to Normal

**Fig. (8):**
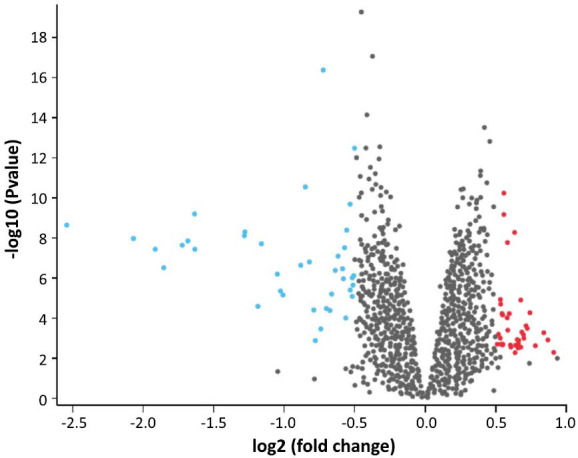
Volcano plot of DEGs. [The volcano plot displays genes that are expressed differentially. The red dots on the graph represent genes that are differentially expressed. Genes that are up-regulated and have a positive logFC value are placed in the upper part of the graph (+0), whereas genes that are down-regulated and have a negative logFC value are located in the lower part of the graph (−0). Genes that did not show significance were represented by black dots].

Our research using the R package identified 76 genes that exhibited differential expression between normal samples and atherosclerotic samples. GSE23746 was obtained from human samples and consisted of 19 samples of normal human monocytes and 76 samples of monocytes from patients. DEGs were determined based on a relevance level that was of *p* < 0.05 and a fold change greater than 0.5. A total of 76 DEGs were discovered from the dataset having both upregulated and downregulated genes (Fig. **8**).

### 3.2 Analysis of Gene Ontology Enrichment

**Fig. (9):**
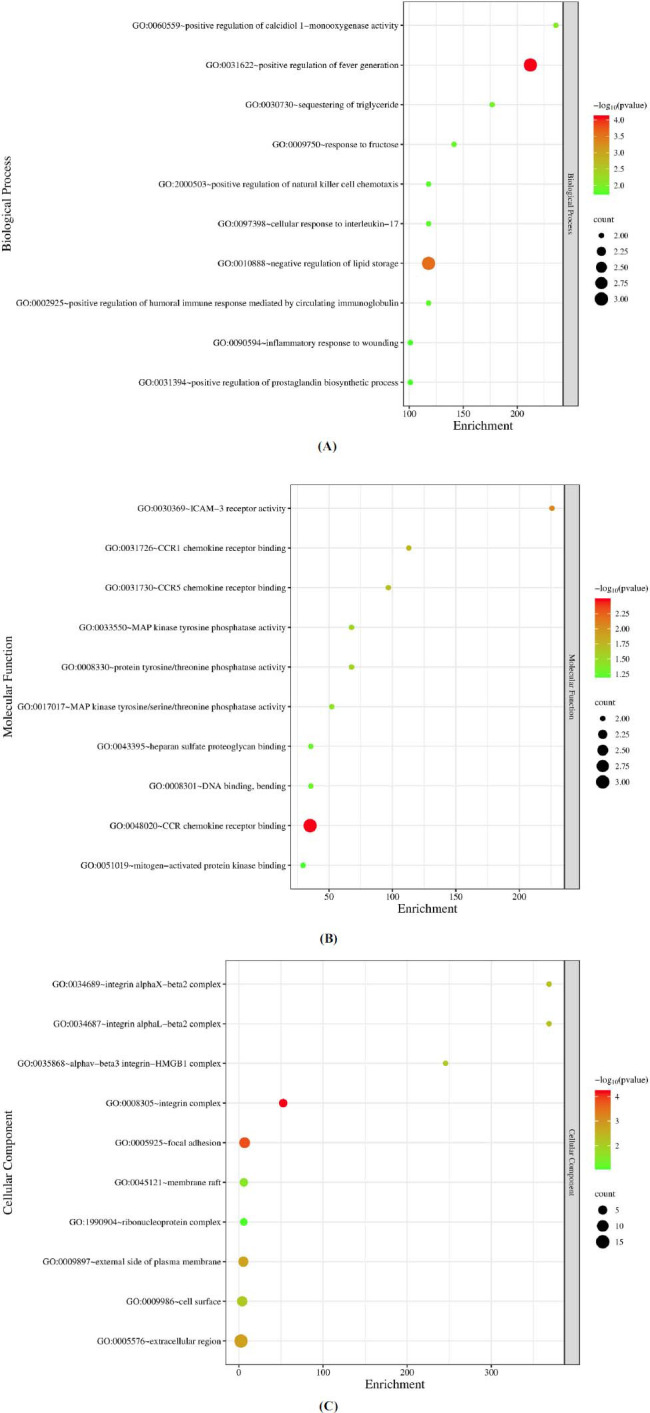
**A**) Biological processes of DEGs. **B**) Molecular functions of DEGs. **C**) Cellular components of DEGs. [Performing GO enrichment analysis on genes that are expressed differentially. **A**) The biological process (BP) analysis reveals that most of the differentially expressed genes are involved in biological regulation. **B**) The cellular component (CC) reveals that the DEGs are involved in several cellular activities. **C**) The molecular function (MF) indicates that those genes are involved in binding and catalytic activities].

GO analysis was recruited to examine the function of DEGs. This section analyzed the biological processes (BP) (Fig. **9A**), molecular functions (MF) (Fig. **9B**), and cellular components (CC) (Fig. **9C**), and of genes that exhibited differential expression patterns. The GO analysis results revealed significant enrichment of changes in BP related to monooxygenase activity, positive regulation of fever generation, sequestering of triglycerides, and response to fructose. Changes in CC were predominantly enriched in the extracellular region, focal adhesion, integrin complex, and cell surface. Furthermore, changes in MF were mainly enriched in chemokine receptor activity, chemokine receptor binding, MAPK tyrosine phosphatase activity, and mitogen-activated protein kinase binding.

### 3.3 A Protein-protein Interaction (PPI) Framework Network and Identification of Hub Genes

**Fig. (10):**
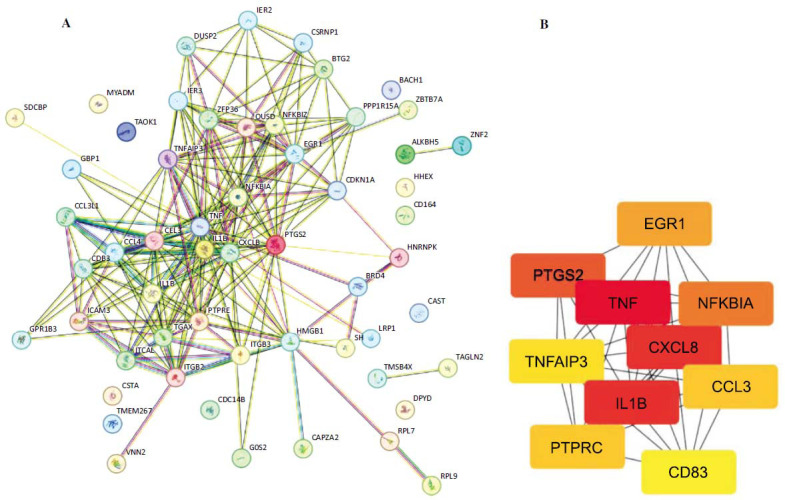
**A**) PPI network of DEGs. **B**) HUB genes Involved in the formation of atherosclerosis. PPI network analysis: (**A**) The PPI network was created using STRING, with 56 nodes and 224 edges. The network was formed using a highly strict minimum needed interaction score of 0.09. (**B**) HUB genes.

All of the DEGs that overlapped each other pointed to a distinct set of interactions and networks. We identified 76 DEGs but only 56 can go for the STRING database to formulate a protein-protein interaction (PPI) network (Fig. **10A**). Ultimately, we discovered a total of 56 nodes and 224 edges in the network. Out of the 56 critical genes, only 10 significant hub genes TNF, IL1B, CXCL8, PTGS2, NFKBIA, EGR1, PTPRC, CCL3, TNFAIP3, AND CD83 were chosen (Fig. **10B**). These genes are mostly involved in the development of atherosclerosis and are also included in the enrichment pathways.

### 3.4 A Protein-protein Interaction (PPI) Framework Network of POU2F3

**Fig. (11):**
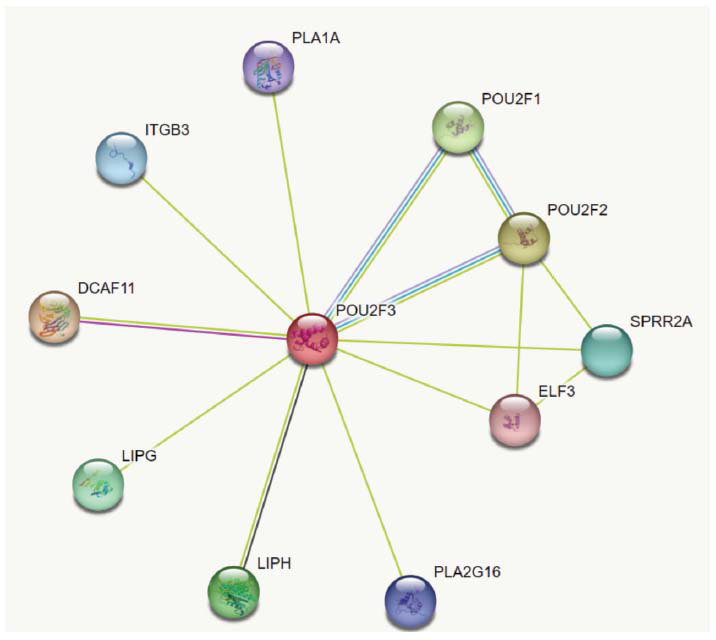
This figure illustrates the network of gene interactions between the 10 most significantly represented genes with POU2F3.

Co-expressed genes that code for proteins may result in closely interdependent biological and molecular functions. Here, using the Search Tool for Retrieval of Interacting Genes and Proteins database, we created a PPI network for genes from important modules [61]. There are a wide variety of biological processes that are affected by the target protein or protein-encoding gene. Some of these contribute to the chain reaction that ultimately results in atherosclerosis. Through this investigation, it was discovered that the targeted gene co-expresses with an additional gene, LIPH, involved in the hydrolysis of phospholipids (Fig. **11**).

### 3.5 A Protein-protein Interaction (PPI) Framework Network of ANGPTL3

**Fig. (12):**
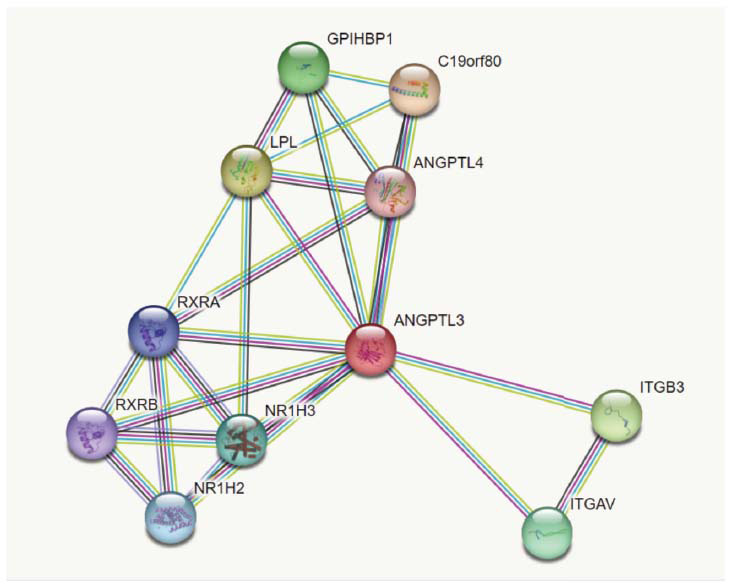
PPI analysis of ANGPTL3.

The clinically relevant secreted ANGPT and ANGTPL proteins perform several functions in both development and disease etiology. According to the findings of our research into the interactions between proteins, there are multiple protein-coding genes were identified that are connected to the ANGPTL3 gene, which serves as the focus of our investigation. One of the genes that contribute to the development of atherosclerosis, ANGPTL4, can be found among those connected to the disease (Fig. **12**). Because some ANGPT/ANGPTL genes-namely, ANGPTL1, ANGPTL2, ANGPTL3, and ANGPTL4 (ANG4)-have been linked to atherosclerosis, Their encoded proteins play a part in aortic lipid deposition, vascular development, remodeling of blood vessels, vascular inflammation, neovascularization, and destabilization of plaque as the disease progresses [62]. Therefore, it is evident from the interaction analysis that the targeted protein has a long history of association with atherosclerosis. And after multiple therapeutic analyses, it will be taken into consideration as a biological marker.

### 3.6 A Protein-protein Interaction (PPI) Framework Network of LCAT

**Fig. (13):**
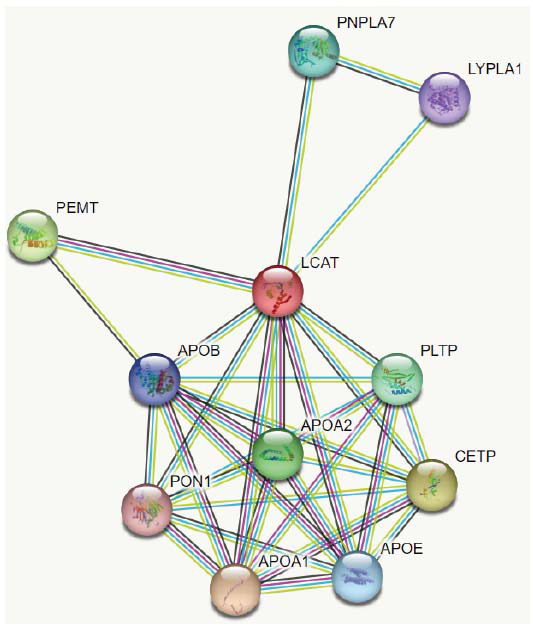
PPI analysis of LCAT gene.

This interaction assessment clearly shows that the LCAT molecule is linked to other protein compounds that have a significant impact on atherosclerosis. These protein molecules are referred to as APOB, APOA1, APOA2, and PON1, respectively (Fig. **13**). These apolipoproteins are essential components that stabilize the structure of lipoproteins, transport cholesterol, and TG throughout the body, serve as ligand molecules for cell surface receptors, and control the activities of enzymes. They are responsible for determining both the physical characteristics and the metabolic destinies of lipoproteins [63]. ApoA-I, for instance, is a significant structural supporter of HDL, activates LCAT, and acts as a ligand molecule for HDL receptors, all of which improve HDL's ability to export HDLC [64, 65]. Cholesterol has a significant function in the body, and ApoB, which acts as the primary framework for LDL and a molecule that binds to LDL receptors, is essential for this process [66]. ApoE has a crucial role in regulating the removal of triglyceride-rich particles from the bloodstream [67]. It is found in high levels in VLDL and chylomicrons and is responsible for transporting fatty acids to tissues where they are used for energy [68]. In addition to these oxidation of LDL plays a crucial role in the development and advancement of atherosclerosis. In the defence of arteries against atherosclerosis, Paraoxonase1 (PON1), an enzyme associated with high-density lipoproteins (HDL) particles, plays a crucial function. It has antioxidant properties and functions as a significant antiatherosclerotic component of HDL by slowing down the oxidation of LDL [69]. Previous studies have consistently shown a negative correlation between PON1 activity and inflammatory responses in many experimental models and clinical settings, including cardiovascular disease [70]. Reduced serum PON1 activity and elevated IL-4, IL-6, and IL-10 are strongly associated [71]. Hence, the PON1 level and the concentration of ILs are highly important in the medical diagnosis of atherosclerosis. Therefore, they can develop the disease due to low HDL levels because of HDL's anti-atherosclerosis action. So, the reduction in LCAT may contribute to atherosclerosis development [72, 73].

### 3.7 Enhanced Analysis of the Pathway

**Fig. (14):**
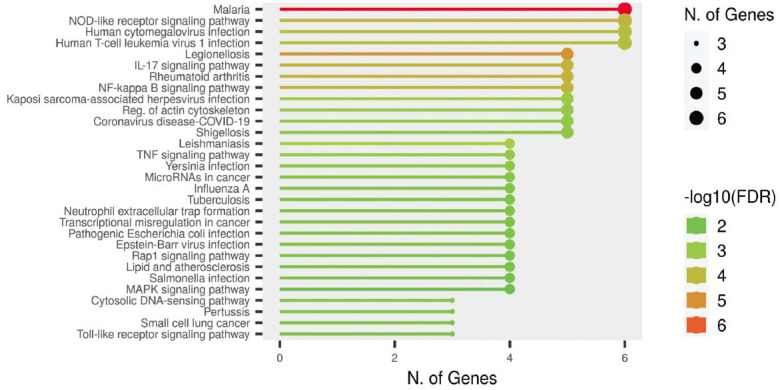
Performing KEGG enrichment analysis on differentially expressed genes (DEGs).

Enrichment analysis of candidate DEGs was conducted utilizing several online databases such as DAVID software and KEGG pathway to gain a deeper insight into their KEGG pathway. The pathway enrichment analysis identified that the development of atherosclerosis involves inflammation, specifically mediated by chemokine and cytokine signaling pathways, such as lipid and atherosclerosis pathways (Fig. **14**).

### 3.8 The Function of Predicted Biomarker

Variations in the circulatory and differentially expressed amounts of microRNAs in blood and other biofluids can serve as valuable non-invasive biomarkers for early detection and prediction of various illnesses [2, 74]. It has also a crucial function in the assessment of diseases which impacted the advancement of pharmaceutical therapies for diseased states [75, 76]. A potential biomarker for atherosclerosis functions acts as a diagnostic tool that aids in the timely identification, tracking, and prediction of the disease's progression. By employing these indicators, medical professionals may get a thorough comprehension of a patient's atherosclerotic condition and make well-informed choices to successfully prevent or treat cardiovascular disease (Table **2**).

## CONCLUSION

Currently, there are a multitude of cardiovascular disease (CVD) biomarkers that provide clinical use as diagnostic, prognostic, or predictive biomarkers. Various biomarkers must undergo rigorous testing to determine their therapeutic usefulness in a diverse range of patients with atherosclerotic cardiovascular disease (CVD) and with varied comorbidities.

The scientific community faces challenges in early detection, addressing risk factors connected to it, and discovering novel compounds with both diagnostic and therapeutic functions. In conclusion, metabolomic-related pathway analysis has been successfully used as a research tool in the study of atherosclerotic disorders. The current research conducted a bioinformatic analysis of differentially expressed genes (DEGs) in paired normal and atherosclerotic samples to identify hub genes associated with and major pathways involved in the development of Atherosclerosis. Selecting hub genes in atherosclerosis is a critical step in understanding the molecular mechanisms underlying the disease and identifying potential therapeutic targets. These genes are highly connected nodes within a gene interaction network, often serving as key regulators of biological processes. In the context of atherosclerosis, hub genes are likely to play central roles in the disease's pathogenesis by orchestrating critical pathways involved in inflammation, lipid metabolism, endothelial function, and plaque formation. Therefore, the objective of this research is to improve our understanding of the molecular mechanism behind atherosclerosis through bioinformatics analysis, potentially providing insights for creating treatments for atherosclerotic patients. In addition to this, it has also been used to assess their severity and provide prognostic information with these problems. Gene Ontology (GO) provides a comprehensive framework to categorize and analyze the roles of genes and their products in the context of biological processes, molecular functions, and cellular components. Using GO pathway enrichment analysis allows for the identification of overrepresented pathways in atherosclerosis. This helps in pinpointing genes that play critical roles in the disease's key pathways, such as cholesterol metabolism and lipid and atherosclerosis pathways.

In comparison to other “omics” technologies like genomics, transcriptomics, and proteomics, an emerging set of metabolomic pathway analysis tools like KEGG and String technologies enable the monitoring of metabolite profiles from biological pathways of these disorders. According to research, phospholipids, Angiopoietin-like 3 protein (ANGPTL3), lecithin cholesterol acyltransferase (LCAT), and the protein encoded by the OCT-1 gene are involved in the pathophysiology of atherosclerosis by promoting LDL infiltration, CD40 acceleration, HDL concentration reduction, and different atherosclerosis-leading processes into the vascular wall and promoting endothelial lesion. Along with those ten hub genes, namely EGR1, PTGS2, TNF, NFKBIA, CXCL8, TNFAIP3, CCL3, IL1B, PTPRC, and CD83, were found to be significantly linked to atherosclerosis. Diagnostic tests for circulating lipoproteins and ceramides are now available, even though these biomarkers are not yet integrated into normal clinical practice. Therefore, there is a robust and ongoing initiative is required that reinforces their function in assessing atherosclerosis.

## Figures and Tables

**Table 1:** Various chemoattractants with corresponding illustrations of each type.

**Types**	**Example**	**References**
Lipid Chemoattractant	Leukotriene B_4_ (LTB_4_)	[16]
	Platelet activation factor (PFA)	[17]
	5-lipoxygenase (5-LO)	[18]
N-formylated Peptides	*N*-formyl-l-methionyl-l-leucyl-l-phenylalanine (FMLP)	[19]
Complement Anaphylatoxins	C5a	[20]
Chemokines	CCL2, MCP-1	[21]

**Table 2:** Function of predicted biomarkers.

**Through HUB Gene Identification**		
**Gene Symbol**	**Gene Name**	**Gene Function**
EGR1	Early growth response factor 1	EGR1 performs important functions in tumor cell proliferation, angiogenesis, invasion, and immune responses.
PTGS2	Prostaglandin-endoperoxide synthase 2	Involved in inflammation and mitogenesis.
TNF	Tumor necrosis factor	This gene encodes a multifunctional proinflammatory cytokine, This cytokine is involved in the regulation of a wide spectrum of biological processes including cell proliferation, differentiation, apoptosis, lipid metabolism, and coagulation.
NFKBIA	Nuclear factor kappa B inhibitor alpha	Include in lipopolysaccharide-mediated signaling pathway, proliferation.
CXCL8	C-X-C motif chemokine ligand 8	The protein encoded by this gene is a member of the CXC chemokine family and is a major mediator of the inflammatory response. The encoded protein is commonly referred to as interleukin-8 (IL-8). IL-8 is secreted by mononuclear macrophages, neutrophils, eosinophils, T lymphocytes, epithelial cells, and fibroblasts.
TNFAIP3	TNF alpha induced protein 3	The encoded protein, which has both ubiquitin ligase and deubiquitinase activities, is involved in the cytokine-mediated immune and inflammatory responses.
CCL3	C-C motif chemokine ligand 3	This locus represents a small inducible cytokine. The encoded protein, also known as macrophage inflammatory protein 1 alpha, plays a role in inflammatory responses through binding to the receptors CCR1, CCR4 and CCR5.
IL1B	Interleukin 1 beta	This cytokine is an important mediator of the inflammatory response and is involved in a variety of cellular activities, including cell proliferation, differentiation, and apoptosis.
PTPRC	Protein tyrosine phosphatase receptor type C	PTPs are known to be signaling molecules that regulate a variety of cellular processes including cell growth, differentiation, mitosis, and oncogenic transformation.
CD83	Cluster of differentiation 83	The protein encoded by this gene is a single-pass type I membrane protein and a member of the immunoglobulin superfamily of receptors. The encoded protein may be involved in the regulation of antigen presentation.
**Through Pathway Analysis**		
Phospholipids	Increases in atherosclerosis	Oxidized phospholipids (oxPLs), which are produced by the oxidation of LDL and the membrane of apoptotic cells attached to Lp(a), build up in areas of persistent inflammation, such as atherosclerotic plaque, and help to accelerate disease.
Angiopoietin-like 3 proteins	Increases in atherosclerosis	Decrease the LPL activity so increase the TG level and cause Atherosclerosis.
Lecithin-cholesterol acyltransferase (LCAT)	Decreases in atherosclerosis	Help in the production of HDL.
Oct-01	Increases in atherosclerosis	Inflammation.

## Data Availability

Gene Expression Omnibus (GEO) database (https://www.ncbi.nlm.nih.gov/geo/) was used for dataset screening in this research work.

## LIST OF ABBREVIATIONS

CAMs: Cell Adhesion Molecules
CETP: Cholesterol Ester Transfer Protein
CHD: Coronary Heart Disease
CSFs: Colony-stimulating Factors
DEGs: Differentially Expressed Genes
EC: Endothelial Cell
GO: Gene Ontology
IFNs: Interferons
ILs: Interleukins
KEGG: Kyoto Encyclopaedia of Genes and Genomes
LDL: Low-density Lipoproteins
MPV: Mean Platelet Volume
PDW: Platelet Distribution Width
PPI: Protein-protein Interaction
TGFs: Transforming Growth Factors
TNFs: Tumor Necrosis Factors
